# An Attempt of Early Detection of Poor Outcome after Whiplash

**DOI:** 10.3389/fneur.2016.00177

**Published:** 2016-10-20

**Authors:** Sebastien Laporte, Danping Wang, Jennyfer Lecompte, Sophie Blancho, Baptiste Sandoz, Antoine Feydy, Pavel Lindberg, Julien Adrian, Elodie Chiarovano, Catherine de Waele, Pierre-Paul Vidal

**Affiliations:** ^1^LBM/Institut de Biomécanique Humaine Georges Charpak, Arts et Metiers ParisTech, Paris, France; ^2^Plateforme d’étude de la Sensorimotricité, Université Paris Descartes, Paris, France; ^3^Institut pour la Recherche sur la Moelle épinière et l’Encéphale (IRME), Paris, France; ^4^FR 3636, Université Paris Descartes, INSERM U894, Paris, France; ^5^Service de Radiologie B, APHP, CHU Cochin, Faculté de Médecine, Université Paris Descartes, Paris, France; ^6^CEESAR, Nanterre, France; ^7^COGNition and ACtion Group (COGNAC-G), Université Paris Descartes – CNRS UMR-MD – SSA, Paris, France

**Keywords:** whiplash outcome, biomechanics, neuro-otology, cognition, tractography, neuropsychological tests

## Abstract

The main concern with whiplash is that a large proportion of whiplash patients experience disabling symptoms or whiplash-associated disorders (WAD) for months if not years following the accident. Therefore, identifying early prognostic factors of WAD development is important as WAD have widespread clinical and economic consequences. In order to tackle that question, our study was specifically aimed at combining several methods of investigation in the same WAD patients at the acute stage and 6 months later. Our longitudinal, open, prospective, multi-center study included 38 whiplash patients, and 13 healthy volunteers matched for age, gender, and socio-economic status with the whiplash group. Whiplash patients were evaluated 15–21 days after road accident, and 6 months later. At each appointment, patients underwent a neuropsychological evaluation, a full clinical neurological examination, neurophysiological and postural tests, oto-neurological tests, cervical spine cord magnetic resonance imaging (MRI) with tractography (DTI). At 6 months, whiplash patients were categorized into two subgroups based on the results of the Diagnostic and Statistical Manual of Mental Disorders as having either favorable or unfavorable progression [an unfavorable classification corresponding to the presence of post-concussion symptom (PCS)] and we searched retrospectively for early prognostic factors of WAD predicting the passage to chronicity. We found that patients displaying high level of catastrophizing at the acute stage and/or post-traumatic stress disorder associated with either abnormalities in head or trunk kinematics, abnormal test of the otolithic function and at the Equitest or a combination of these syndromes, turned to chronicity. This study suggests that low-grade whiplash patients should be submitted as early as possible after the trauma to neuropsychological and motor control tests in a specialized consultation. In addition, they should be evaluated by a neuro-otologist for a detailed examination of vestibular functions, which should include cervical vestibular evoked myogenic potential. Then, if diagnosed at risk of WAD, these patients should be subjected to an intensive preventive rehabilitation program, including vestibular rehabilitation if required.

## Introduction

Whiplash is usually defined as an injury of the neck, which most often occurs following car rear-end collision. In the present study, they consist mostly in soft tissues injuries since we excluded patients with fractures and dislocations. The main concern with whiplash is that a large proportion of these patients experience disabling symptoms for months if not for years following the accident. A consultation paper presented to the English Parliament in 2012^1^ illustrates the magnitude of the societal problem raised by the number and costs of whiplash claims. Between 2006 and 2010, the number of reported road traffic accidents (RTAs) fell by around 20%. During the same period, the number of claims for personal injury arising from RTAs rose by around 60%, and around 70% of RTAs personal injury claims are for whiplash injuries. According to the Association of British Insurers, more than 1,500 whiplash claims are made in the United Kingdom every day, costing the insurance industry more than £2 billion a year.

The whiplash problem has generated no less than 566 review articles on whiplash since 1964, which summarize 3,266 papers since 1952. In that context, we will mostly quote reviews in the following assessment of what is already known about whiplash. Several key aspects of whiplash have been investigated.

First, although the majority of acute patients with whiplash-associated disorders (WAD) show no visible physical damage to the neck, up to 50% of them develop chronic pain and that is their main complaint. Contrary to common believe, there is no clear evidence that compensation and its related processes lead to worse conditions following whiplash (1). Whiplash injury would rather induce peripheral sensitization (hypersensitivity of the peripheral nociceptors) and central sensitization (hyper excitability of population of neurons in the central nervous system), which could persist for months and even years past the acute phase of the whiplash. Indeed, recent reviews (2, 3) pointed to clinical signs to evaluate WAD, which suggest a role of central sensitization in chronic whiplash associate disorders: persistent pain complaints, local and widespread hyperalgesia, referred pain, allodynia, decreased spinal reflex thresholds, inefficient diffuse noxious inhibitory control activation, and enhanced temporal summation of pain.

Second, previous studies showed several factors appear to increase the risk of subsequent chronic whiplash disorders (4–6), such as experiencing a pain condition pre-accident, low-income, poor self-rated health, diffuse musculoskeletal pain, somatic symptoms, use of multiple health services, high use of medications, symptoms of anxiety, and high level of catastrophizing. The Carstensen review (7) pointed to other types of predisposing factors on whiplash recovery: being unemployed before the collision, low education level, being a female, accumulation of distress as opposed to a single distress factor (e.g., depression) predicted poor recovery. Also, the stress response may contribute to the development of a chronic WAD (8, 9). Finally, Walton in his 2013 review paper (10) pointed to high pain intensity (greater than 5.5/10 on the standard pain scale), female gender, report of headache at inception, lower education (less than postsecondary), high neck disability index, WAD grade 2 or, pre-injury neck pain, report of low back pain at inception. Furthermore, several variables did not appear to be predictor of chronic WAD: impact direction, seating position, awareness of impending collision, head rest in place, older age, vehicle stationary when hit, regular physical activity (11).

Third, 70% of chronic whiplash patients (CWP) complain of dizziness and unsteadiness (12). On the motor side, reduced cervical mobility, disturbed kinesthesia and altered neck muscles activity are often present at the acute stage and they persist over time in the moderate/severe whiplash groups of patients (13). Several reviews also point to dysfunctions of postural control in CWP (14–16). On the sensory side, within 1 week of their trauma, 40% of acute whiplash patients (AWP) report dizziness and 10% of them develop later otological symptoms, such as tinnitus, deafness, and vertigo (17). Vestibular deficits may be at play because some AWP and CWP exhibit nystagmus, abnormal gain of the vestibulo-ocular, vestibulo-collic and cervico-ocular reflexes, and abnormal values of the vestibular evoked potentials. The vestibular deficits can be uni- or bilateral. The clinical syndromes of whiplash patients can also result from abnormal neck somatosensory information. In particular, the sensitivity of the neck muscles spindle could be affected by ischemic or inflammatory events and degenerative changes. Furthermore, being at the convergence of the somatosensory, vestibular, and visual systems on several central nervous system (CNS) structures, abnormalities of one of more of these subsystems can lead to the oculomotor, cephalic, and postural syndrome reviewed in Kristjansson and Treleaven (18). Therefore, a detailed investigation of the sensorimotor control seems warranted for all patients with severe neck pain, including an investigation of postural and oculomotor control, the cervical joint position sense and a neuro-otological examination (19).

Finally, as reviewed by Hol (20), computed tomography (CT) is increasingly replacing plane radiography in acute WAD patients. It is used not only to detect cervical fractures, dislocation or displacement, but also to diagnose injury in the paravertebral soft tissue, disk, and cord. Additionally, in the lowest risk group of acute WAD patients without neurological deficits and in chronic WAD patients, the role of imaging is still controversial. To the best of our knowledge, tractography had not yet been tested in whiplash patients. In summary, whiplash offers a complex picture and, as underlined above, the main problem is that about 50% of patients experience chronic symptoms with considerable direct and indirect costs.

In that context, early detection of the whiplash patients at risk of developing WAD is important because it would allow not only designing better preventive treatment for high-risk individual (21) but also paving the way for prevention [see, for instance, Ref. (22)]. It remains that the great heterogeneity of the clinical investigations in these patients has resulted in disparate if not contradictory results on that matter. Moreover, a vast majority of studies focused on a limited numbers of WADs. Therefore, the mechanisms behind the transition from acute to chronic WAD remain to be elucidated and in particular the evaluation of the impact of multiple risk factors in a single patient. In order to tackle this problem, we set out to combine several methods of investigations in a group of WAD patients at the acute stage of whiplash and we have repeated these observation 6 months later. The patients had whiplash injury grades I, II, and III, according to the Quebec Task Force Classification of WAD (23). Our study encompassed several types of investigations: a psychologist interviewed participants. Then, a neurologist conducted a detailed clinical examination and two neurophysiologists submitted the patients to more specialized test concerning gaze and postural control. A neuro-otologist conducted a thorough clinical examination and clinical tests of vestibular function. Finally, the patient underwent magnetic resonance imaging (MRI) of the neck, including a tractography (DTI) study of the descending tract in the spinal cord.

## Materials and Methods

### Subjects

A longitudinal, open, prospective, multi-center study was performed. Two groups were assessed: patients diagnosed with whiplash and healthy volunteers.

Thirty-eight patients with acute WAD were assessed at the early stage, within 15–21 days following injury and then 6 months after the accident. The sample of acute WAD patients was composed of 22 women and 16 men, aged 19–56 years old (mean age 35 ± 11 years old). They were recruited from the emergency departments of two university hospitals (Hôpital Bicêtre and Hôpital Cochin, Paris, France). They all complained about neck pain in the first 48 h after the collision. Table 1 gives the number of volunteers and patients for each specific tests at 15–21 days (D15–21) and at 6 months (M6). The inclusion criteria for the WAD patients were (a) to be subjected to WAD classified as grade I, II, or III according to the Quebec Task Force (23), (b) to have brain injury associated and/or initial loss of consciousness for more than 30 min after the collision, and (c) to have an initial Glasgow Coma Scale (GCS) score of 15, 30 min after the collision.

**Table 1:** **Number of volunteers and whiplash patients included in the present study at 15–21 days (D15–21) and at 6 months (M6) after the whiplash**.

	Volunteers	Whiplash patients	
		D15–21	M6
Neuropsychological analysis	13 (7F/6M)	38 (22F/16M)	37 (21F/16M)
Kinematics analysis	13 (7F/6M)	38 (22F/16M)	37 (21F/16M)
Otoneurorogical analysis	8 (4F/4M)	38 (21F/16M)	17 (9F/8M)
Imagery analysis		22 (12F/10M)	22 (12F/10M)
Early detection analysis	13 (7F/6M)	17 (9F/8M)	17 (9F/8M)

The exclusion criteria were intubation, ventilation, or sedation on arrival at hospital; a spinal cord injury with neurological symptoms or a disabling polytrauma with at least one injury considered life-threatening; a whiplash after a suicide attempt; psychiatric, psychological or otorhinological disorders that were either disabling or might interfere with follow-up; psychotropic medication intake at the time of injury; a history of hospitalization in a specialized psychiatric unit and/or sick leave for psychological reasons; a history of severe head injury; a progressive neurological disease; a drug addiction; under guardianship; and a contraindication to MRI.

Thirteen healthy subjects (seven women and six men, mean age 41 ± 17 years old) were included after a specific medical examination and reviewed of a questionnaire they were asked to fill out about their medical history and neck pain. The questionnaire was designed to confirm that none of the control subjects had history of neck disorders.

This study was approved by the ethical committee of the Pitié-Salpétrière university hospital (Paris, France). All subject signed an informed consent form before they underwent any study procedures (ID RCB 2009-AO1002-55).

### Neuropsychological Factors

#### Protocol

The patients attended two testing sessions at the Hopital Cochin, Université Paris Descartes. The sessions lasted approximately 1 h. Cognitive tests and questionnaires were done in a pre-determined order. Patients were given as much break time as they desired along the session. The first sessions took place within 15–21 days following the whiplash. It began with an interview in order to assess the case history of the patient and the aftermath of the accident on patients’ life. In the second session, within 6 months following the whiplash, another interview was conducted to assess if patients had recovered.

Data were collected for demography, circumstances of the accident, and medical history. Standardized and classical psychopathological scales, quality of life (QoL) questionnaires, and visual analog scale (VAS) are described below.

#### Quality of life

Global QoL was evaluated using a visual scale on which subjects had to indicate their degree of satisfaction in life overall (all areas combined) for the 15 days prior to the evaluation. An in-depth evaluation was performed using the Quality of Life After Brain Injury (QOLIBRI) questionnaire (24), which is specifically adapted to brain injury patients and is designed to evaluate QoL in all domains of daily life (cognitive, physical, social, emotional, and personal) and validated in French.

#### Pain

The intensity of pain was evaluated using a VAS (maximum value of 10). The “questionnaire Saint Antoine” was also used as it is adapted to whiplash patients and evaluates 16 major complaints reported in post-concussion symptom (PCS), including the intensity of the complaint. The Saint-Antoine Pain Questionnaire (QDSA: Questionnaire Douleur Saint-Antoine) (25) is a French version of the McGill Pain Questionnaire (26) and a VAS.

#### Anxiety and Post-traumatic Stress Disorder

A mood evaluation scale was used to evaluate the presence of anxiety [State-Trait Anxiety Inventory A self-evaluation scale (STAI-A Forms Y-A and Y-B, French version)]. Post-traumatic stress disorder (PTSD) was assessed using the French version of the impact of events scale-revised (IES-R) (27). This scale was designed to assess three dimension of the PTSD: avoidance, intrusion, and hyperarousal.

#### Outcome

We have selected subjective measures to assess the outcome 6 months post-accident. A patient-centered approach in assessing “recovery” in injured persons has been recommended in a prior research (28). We used the VAS. Consistent with Carstensen et al. (29), pain scores less than or equal to 30 mm are considered as minor, while scores above 30 mm are considered as severe pain. It resulted in splitting patients in two groups at 6 months, based on the VAS results: the chronical (Group C) and non-chronical (Group NC) groups.

### Head and Trunk Kinematics

Head and trunk kinematics were recorded using an active 3D Motion tracking system (Coda CX1 Scanner Units, Charnwood Dynamics Ltd. Rothley, Leicester, UK, 1996). Eight Coda active markers were attached to the head–neck segment: four on the head (each of the tragus, nasion and the occiput projection) and four on the trunk (bilateral acromion processes, the sternal notch and C7 projection). The markers were powered by light-weight battery packs on the marker drive boxes and powered from them. The markers were glued to the subjects’ skin using double-sided hypoallergenic sticky tape. Their 3D positions were measured in real time with a sampling frequency of 200 Hz. Based on these markers, a trunk anatomical frame was calculated (30) where *X* is the posterior-anterior axis, *Y* the medial-lateral axis pointing to the left, and *Z* the caudo-cranial axis.

Cervical motor dysfunctions at the acute and chronic stages after whiplash trauma had been investigated for long. They are still routinely used to reveal the presence of cervical motor dysfunctions to follow their course and to assess their predictive value for long-term recovery [see Daenen et al. (13) for a review]. Head repositioning and more generally cervical kinesthesia had also been studied in detail in whiplash patients (31–35).

#### Range of Motion Test

Cervical movements were performed in standing posture, eyes open. The standing posture was chosen in order to capture the global behavior of the equilibrium, even if the study is focused on the head–neck complex. The self-chosen “neutral” position of the subject was set as a reference (zero). A laser pointer fixed on the top of the head of the subject and a target projected on the wall helped the subject to accurately return to this “neutral” position. Subjects were asked to perform three maximal active neck movements in flexion–extension (FE), left–right side bending (SB), and left–right axial rotation (AR) at a self-selected speed. For each movement, the subject started and finished in the neutral position. Each movement was repeated three times with a short break between each occurrence. Rotations were performed randomly.

Relative angular displacement of the head in the anatomic thoracic plane was calculated using mobile axes with sequences YZ′X″ for FE, XZ′Y″ for SB, and ZX′Y″ for AR. The calculated angles were smoothed by a robust locally weighted regression (36), which acts as a low pass filter without distorting the results (37). Maximal ROM for primary motion (PM) and coupled movement (CM) was calculated from the maximum value of three trials.

#### Head to Neutral-Head-Position Test

The Head to NHP was performed in standing posture, blindfolded. Subjects were asked to return in neutral position as accurate as possible after a sub-maximal movement in the following directions: left AR, right AR, and extension. The laser and a target projected on the wall helped the operator to accurately passively replace the head of the subject into “neutral” position after each movement. The repositioning error was evaluated with the calculation of two error types: (a) the absolute error $(\text{AE})=\Sigma |{\text{Error}}_{\text{i}}|/N$, and (b) the root mean square error $(\text{RMSE})=\sqrt{{\text{CE}}^{2}+{\text{VE}}^{2}}$ with constant error $(\text{CE})=\Sigma {\text{Error}}_{\text{i}}/N$, and variable error $(\text{VE})=\sqrt{\sum {\left({\text{Error}}_{\text{i}}-\text{CE}\right)}^{2}}/N$ (38, 39).

#### Head Pursuit Movements

Subjects were asked to make additional movements to track the displacement of a visual target with the laser pointer attached to the head. The visual target was projected on to a large screen at a distance of 2.25 m. The target was made to move along one of three different trajectories, a linear left-right sinusoidal movement of 2.15 m peak-to-peak that could require ±25° yaw rotations of the head, a linear up-down sinusoidal movement of 1.42 m peak-to-peak that required ±20° pitch rotations of the head, and an lying-eight-figure movement, that required a ±20 movement in yaw and a ±15° movement in pitch in order to accurately follow the target with the dot of the laser pointer on the screen. For the linear motions, the target moved for three cycles at a total duration of 10 s per cycle. For the lying-eight, a target followed the figures, moving at three different speeds: 30, 20, and 10 s, respectively, for the slow, medium, and fast speeds. Subjects were instructed to follow the target by moving the head only.

Residual movements of the trunk could not be excluded. Rotation angles of the head and trunk were computed around the laboratory *Z* and *Y* axes, corresponding to yaw and pitch rotations. For the lying-eight-figure movements, we computed the mean of the rotational acceleration root mean square (RMS) of the trunk and the head around each axis (TRUNKaj and HEADaj, with j = S for slow, M for medium, F for fast); these were used to categorize the smoothness of the head and trunk movements. For linear pursuit movements, we refined the analysis to focus on movements of the trunk that were correlated to the movements of the head, ignoring spurious movements or constant biases in the trunk angular position. To this end, we computed the cross-correlation between rotations of the head and of the trunk and divided these values by the autocorrelation of the head rotation with itself to normalize across subjects. This dimensionless (RMS_Yaw and RMS_Pitch) index increases with the increase correlation of the movements of the trunk with the head movements, and decreases when there little or no correlation at all. These two indexes are then linked with the global equilibrium of the volunteer/patient.

We also examined the symmetry of rotational movements of the head. For a sinusoidal movement of the target with amplitude *A* and frequency *f* [*P*_target_ = *A* cos (f*t/*2π)] projected on the screen at distance *D* from the subject, the ideal head rotation is described by the equation θ = tan^−1^(*A* cos(f*t/*2π)/*D*), which is symmetric with respect to 0. For integral whole number of cycles, the mean and median angle computed over the entire trajectory should be the same. If, however, the subject truncated the oscillation more in one direction than the other, the distribution of time spent at various distances from the midpoint in each direction would be different. The extent of the motion from the median value to the extremes could differ for each direction, depending on how much the movement of the head was truncated in each direction. Then, we computed two indicators of asymmetry. The first was the absolute value of the mean minus median head angle across the three cycles of the target movement. The second was the absolute difference between distance between the 5 and 50% quantile values and the distance between the 50 and 95% quantile values, with the idea that the 5 and 95% quantile values provide a more robust measure of movement amplitude in either direction than the peak value.

#### Statistical Analysis

Pursuit results, principal and CMs RoM, as well as NHP and posture were compared separately across groups using an ANOVA, Newman–Keuls *post hoc* tests were used in subsequent analyses. The level of statistical significance was *p* < 0.05.

### Otoneurorogical Tests

The otoneurorogical tests required an additional session in an ENT (Ear Nose Throat) department; therefore, it was not accepted by all the control subject and patients. Consequently, this part of the study included the following subset:

– A control group (*n* = 8, 4F/4M, mean age: 41, 5 ± 17.1)
– A large group of patients at the early stage (*n* = 38, 22F/16M, mean age: 36.6 ± 10.3, min–max: 20–56), time post-injury: 29.8 ± 15.2 days (11–73).
– A smaller group of patients tested at the chronic stage (*n* = 17, 9F/8M, mean age: 35.7 ± 9.1, min–max: 22–50), time post-injury: 235.0 ± 48.6 days (141–313).

#### Audiometric Tests

Hearing loss was appreciated using pure-tone audiometry. The pure-tone threshold average (PTA) for tones at 500 Hz, 1 kHz, and 2 kHz was used as an indicator of hearing loss. Tympanometry and stapedial reflex were carefully evaluated to exclude patients with a conductive (even slight) hearing loss to avoid misinterpretation of air-conducted sound vestibular evoked myogenic potential (ACS VEMPs).

#### Vestibular Tests

The vestibular function was assessed using the following tests:

– Evaluation of the horizontal canalar function using videonystagmography (VNG). We first search for spontaneous ocular nystagmus in darkness. The presence of induced nystagmus was investigated using vibrations at 100 Hz of both mastoid and head shaking test. Finally, caloric and head impulse tests followed if spontaneous or induced nystagmus were found.
– Evaluation of the otolithic inferior and superior vestibular function by means of cervical vestibular evoked myogenic potentials (cVEMPs) and ocular vestibular evoked myogenic potentials (oVEMPs), respectively.
– Evaluation of the equilibrium function using Equitest©.

#### Caloric Testing

Caloric tests were performed in case of abnormal VNG (head shaking positive, vibratory positive, spontaneous nystagmus in sitting, and supine position). Caloric tests were performed using closed loop sequential bithermal irrigation with water at 30 and 44°C and VNG. Percent of canal paresis (CP) was calculated using Jongkees’ formula: CP = 100 × [(UW + UC) − (AW + AC)]/(UW + UC + AW + AC), where UW, UC, AW, and AC are velocity of the induced ocular nystagmus obtained on the unaffected and affected sides, with warm and cold water, respectively. A value of CP greater than 20% was regarded as an abnormal decrease on the affected side.

#### VEMPs Testing

Two types of VEMPs are now widely used in clinics. cVEMP, which is recorded in the sternocleidomastoid muscle reflects sacculo-collic reflex. oVEMP, recorded below the lower eye lid and predominantly reflects utriculo-ocular reflex. VEMPs play key roles not only for assessment of vestibular [see, for review, Ref. (40)], neurological diseases [see, for review, Venhovens et al. (41)] and elderly patients [see review of Piker et al. (42)].

Vestibular evoked myogenic potentials were recorded with a Nicolet Viking 4 apparatus (Nicolet Biomedical Inc., Madison, WI, USA) with a 4-channel averaging capacity. The clicks (0.1 ms rarefactive square waves of 105 dB nHL) and short tone burst (STB, 500 Hz, 102 dB nHL, 128 dB SPL, rise/fall time 2 ms, plateau time 2 ms) were presented through calibrated TDH39 headphones. BCV stimuli (bone-conducted vibration) were 500 Hz STB (rise/fall time = 2 ms and plateau time = 2 ms). They were delivered by a hand-held Bruel and Kjaer (Naerum, Denmark) Mini-Shaker 4810, fitted with a short bolt (2 cm long, M4) terminating in a Bakelite cap 1.5 cm in diameter, which was the contact point for the stimulator on the subject. The Mini-Shaker weighs approximately 1 kg and the weight of the shaker was used to standardize the force applied to the subjects. The Mini-Shaker was calibrated using a sound level meter (Bruel and Kjaer 2250, calibrated to read 0dB at 1 μV) and an artificial mastoid (Bruel and Kjaer 4930). The voltage drive peak to peak used for 500 Hz STB was 12V, corresponding to a 135 dB FL.

Cervical vestibular evoked myogenic potentials were recorded from the sternocleidomastoideus (SCM) muscles ipsilateral to the stimulated ear in response to AC STB and click stimuli. Patients lay supine on a bed and were asked to lift their head off the pillow and orient it contralaterally to the ear tested to activate maximally the SCM muscle ipsilateral to the stimulation. EMG activity of the SCM was monitored on a display to ensure that sufficient muscle activation was maintained (>150 μV). Latencies of the two early waves (P13 and N23) of the cVEMPs were measured in ms, and the peak-to-peak amplitude between P13 and N23 waves was measured in microvolt.

Ocular vestibular evoked myogenic potentials were recorded from the extraocular muscles contralateral to the stimulated ear in response to AC STB, to BCV at AFz (to the midline forehead at the hairline, the skull location identified as Fz or AFz) and at the mastoid (just behind the pinna of the ear on the mastoid process with the Mini-Shaker held perpendicular to the skin surface). Patients lay supine on a bed and were asked to direct their gaze at a target located 1 m away at an elevation of 25°. The active self-adhesive electrode was placed on the orbital margin, 0.5 cm below the lower eyelid and referred to a parallel electrode below it (~2 cm below the lower eyelid). We measured the peak latencies in milliseconds and the peak-to-peak amplitude in microvolts of the two early waves (n1 and p1). If the peak-to-peak n1–p1 amplitude was smaller than 2 μV, the response was considered as absent. The percentage of VEMP asymmetry in patients with unilateral vestibular lesions was measured by calculating the evoked potential ratio (EPr) as follows: EPr = 100 × [Au − Aa]/(Au + Aa), where Au is the P13–N23 or n1–p1 peak-to-peak amplitude from the unaffected side, Aa is the P13–N23 or n1–p1 peak-to-peak amplitude from the affected side, and [Au − Aa] the absolute value of (Au − Aa).

Patients with a positive response from the intact side were defined as responder subjects. Patients with no response on either side were considered as non-responders (NR). In responder subjects, the response was defined as normal if the EPr was below the threshold value and abnormal if EPr was above the threshold. This threshold was calculated as the mean EPr for the control group + 2SD for the five VEMP tests.

#### Equitest

Computerized dynamic posturography combines a moving force platform with visual and proprioceptive stimuli to determine the relative importance of various sensory inputs critical for balance control. The sensitivity and specificity of this test to assess balance in vestibular patients [see reviews of Furman (43) and Di Fabio (44)], in seniors at risk of fall [see review of Whipple et al. (45)] is well establish since 20 years and continue to be use in new fields to assess post flight postural ataxia in astronauts (46).

The Equitest enabled to quantify balance disorders and to assess whether the vestibular, visual or proprioceptive input were used correctly. Postural control was assessed by measuring the displacement of the vertical forces exerted by the patient’s feet under six conditions:

– maintaining balance with eyes open – C1;
– maintaining balance with eyes closed – C2;
– maintaining balance with eyes open with controlled vision (displacement of visual surround according to changes in the patient’s center of gravity) – C3;
– maintaining balance with eyes open with controlled proprioception (the platform moves in the sagittal plane according to the changes in the patient’s center of gravity) – C4;
– maintaining balance with eyes closed with controlled proprioception – C5;
– maintaining balance with eyes open with controlled proprioception and vision – C6.

The final score was given for each condition in three consecutive tests. It allows the physician to ascertain the sensory preference used by the patient to maintain balance. Patients making little or poor use of their vestibular information, for example, show a poor performance or fall under conditions C5 and C6. A composite score taking into account all the conditions was given. If this score was abnormal, the equilibrium function was considered as abnormal and the sensory scores were study.

#### Statistical Analysis

Statistical analyses were performed using SAS 9.3 statistical software. For comparisons of numerical data, we used a non-parametric Wilcoxon test or a *t*-test depending on to the normality distribution of the data. For comparisons of the distribution (percentage), we used an exact Fisher test or a χ^2^ test based on the expected effects. A threshold of *p* < 0.05 was considered as significant.

### Imagery

#### Subjects

Detailed MR imaging of the cervical spinal cord and of the soft tissues was obtained in 22 whiplash patients early after and at 6 months after whiplash.

#### Magnetic Resonance Imaging

Cervical neck imaging was performed at the Dept Radiology B, Hôpital Cochin, Université Paris Descartes. We used a 1.5-T Siemens Magnetom Avanto MRI equipped with Neck Matrix Coil. Sequences included sagittal FSE T1-weighted imaging for study of signs of muscle atrophy. Axial FLAIR and FSE T2-weighted sequences were used to study presence of discovertebral and spinal cord traumatic lesions including micro-bleeds. Dynamic MRI was performed with the patient’s head and neck in neutral (comfortable supine position) and in 20° of extension (with a foam roll under the patient’s neck). Both STIR and T2 images were obtained in neutral and extended positions. Comparison of the spinal cord space between neutral and extension positions allowed assessment of presence of dynamic cervical instability.

Spinal diffusion tensor imaging (DTI) was also performed in order to quantify spinal cord structure at cervical levels C1–C6. The sensitivity encoding (SENSE) single shot echo-planar imaging (EPI) sequence with SENSE factor 2 was used to reduce distortions. For the sagittal diffusion-weighted sequence, 25 non-collinear gradient directions were applied with two *b*-values (*b* = 0 and 900 s/mm^2^; TR/TE 2,000/95 ms; field of view 18 cm, image matrix 128 × 128; 12 slices with slice thickness of 3 mm; slice gap = 0; voxel size 1.4 mm × 1.4 mm × 3 mm). Spatial presaturation bands were applied both anterior and posterior to the vertebral column to reduce ghosting from fat lying outside the spinal column, and a standard three-dimensional (3D) shim was applied to ensure the best field homogeneity. The sequence was repeated four times, and took 4 min 26 s in total to be executed. DTI images were averaged across the four acquisitions before analysis to increase the signal-to-noise ratio. Imaging parameters were similar to those used in previous DTI studies of the cervical spinal cord (47, 48).

Anatomical MRIs were assessed by an experienced radiologist. DTI images with motion or distortion artifacts were excluded prior to calculation voxel by voxel parameters, using MedINRIA software.^2^ Fractional anisotropy (FA), apparent diffusion coefficient (ADC), axial diffusivity (AD), and radial diffusivity (RD) values were obtained for regions of interest (ROIs) covering the entire spinal white and gray matter were drawn on *b*0 images (47, 48). FA, ADC, AD, and RD were, thus, obtained for ROIs covering the whole of the spinal cord at C1–C6 levels. To limit the partial volume effect [inclusion of voxels containing cerebral spinal fluid (CSF)], it was verified that ROI placement on the b0 image did not contain voxels extending into CSF (voxels covering both were removed). Only voxels with diffusion predominantly in a craniocaudal direction on FA color maps were included.

### Looking for an Early Detection of Poor Outcome after Whiplash

The aim of this part was to identify what association of symptoms and tests described in that study could predict the risk of passage to chronicity in the whiplash patients. As previously mentioned, whiplash patients were split in two groups, based on VAS, which was used to evaluate pain at 6 months: the Chronical (Group C) and non-chronical (Group NC). This part of the analysis was conducted only on the 13 volunteers and the 17 WAD patients who completed all the tests at M6.

One way Kruskal–Wallis test was used to identify the early differences between Group C and Group NC of Whiplash patients and the group of volunteers (Group V) for all the calculated parameters of the head and trunk kinematics and otoneurogical tests at D15–21. A difference of *p* < 0.05 was considered as significant.

## Results

### Neuropsychological Factors

At 6 months post-injury 16 patients (43% of all patients who completed the second session) met our criteria of a chronic whiplash. The group was composed of 6 men and 10 women, and all chronic WAD patients had a college degree.

The results of the statistical analysis first revealed that chronic patients are significantly older than non-chronic patients (44.1 vs. 32.1 years old, *p* < 0.05). Chronic patients also presented, at the early stage, significantly higher levels of pain than non-chronic patients, for the VAS (4.0 vs. 1.7, *p* < 0.05) and the QDSA (17.5 vs. 31.6, *p* < 0.05). Chronic patients have significantly higher PTSD scores on IES-R (31.6 vs. 17.3, *p* < 0.05) than non-chronic patients. The results also indicate that chronic patients have significantly higher state anxiety, as measured by the STAI-YA at the acute stage (45.4 vs. 35.4, *p* < 0.05). Finally, the QoL evaluated with the QOLIBRI is significantly lower in chronic patients than in non-chronic ones (54.7 vs. 76.9, *p* < 0.005).

### Head and Trunk Kinematics

#### Voluntary Head vs. Trunk Movements

##### RoM Primary Motion

At the acute stage, results of head–neck PM showed reduced maximal amplitude in sagittal (81° ± 18° vs. 95° ± 10°; *p* < 0.05) and axial planes (124° ± 23° vs. 139° ± 16°; *p* < 0.05) for WAD (between D8 and D21 post-trauma) compared to controls. No between-groups difference was observed in the coronal plan. Moreover, WAD subjects complaining of pain had a significant larger loss of AR RoM (114° ± 26°, *p* < 0.05) than pain-free patients (132° ± 17°).

At the chronic stage, we noticed a significant increase (*p* < 0.05) of the AR RoM for the pain-free patients (+7° ± 5°), who on average regained control values (139° ± 38°). By contrast, we observed a significant decrease (−35° ± 12°; *p* < 0.05) for the painful patients (88° ± 38°). Finally, whiplash patients regain values close to the ones of the healthy group for flexion–extension movement (92° ± 13°).

##### Coupled Movement

During side bending PM, our results showed higher AR CM in WAD compared to controls (37° ± 20° vs. 23° ± 13°; *p* < 0.05). In contrast, during AR, although the side bending CMs were significantly larger in WAD patients than in control, the difference was negligible. Finally, CMs were absents in both groups for flexion/extension movements.

##### Head to Neutral-Head-Position

At the acute stage, results of the NHP test did not show any difference between WAD and controls, irrespective of motion direction (Table 2). No difference was found for WAD 6 months after the trauma.

**Table 2:** **Repositioning absolute errors (AE) and root mean square errors (RMSE) in neutral position after head–neck extension (EXT), right (R) and left (L) axial rotation (AR) in whiplash patients at early (WAD D15–21) and chronic (WAD M6) stages compared to the control group**.

	EXT (°)		R AR (°)		L AR (°)	
	AE	RMSE	AE	RMSE	AE	RMSE
WAD D15–21	2.6 ± 1.8	3.9 ± 2.5	2.6 ± 1.3	3.9 ± 2.1	2.6 ± 1.5	4.0 ± 2.3
WAD M6	2.6 ± 1.8	4.1 ± 2.2	2.4 ± 1.5	4.3 ± 2.8	2.7 ± 1.5	4.0 ± 2.2
Control	2.2 ± 1.1	3.2 ± 1.2	2.3 ± 1.6	3.7 ± 1.9	2.2 ± 1.1	3.8 ± 2.1

*Mean ± SD*.

#### Head Pursuit Movements

##### Extent and Asymmetry of the Linear and the Horizontal Figure Eights Pursuit Movements

We found no significant difference between patients and controls for the horizontal movements at the acute and chronic stages, but there was a significant difference in the extent of vertical movements. But even though the total extent of horizontal movements were similar between the two groups of subjects, by both measures of asymmetry (difference between mean and median; relative size of quantile intervals) some, but certainly not all (6 out of 38), showed asymmetry values for horizontal pursuit movements well above the range observed in control subjects. A bootstrap calculation showed that the distributions of asymmetry values for patients and controls were significantly different at the *p* < 0.05 level. That is, some patients likely reduced pain on that side by minimizing the trajectory on one side. This was not the case for vertical pursuit movements of the head where patients and subjects showed close distributions of asymmetry indicators.

**Figure 1:**
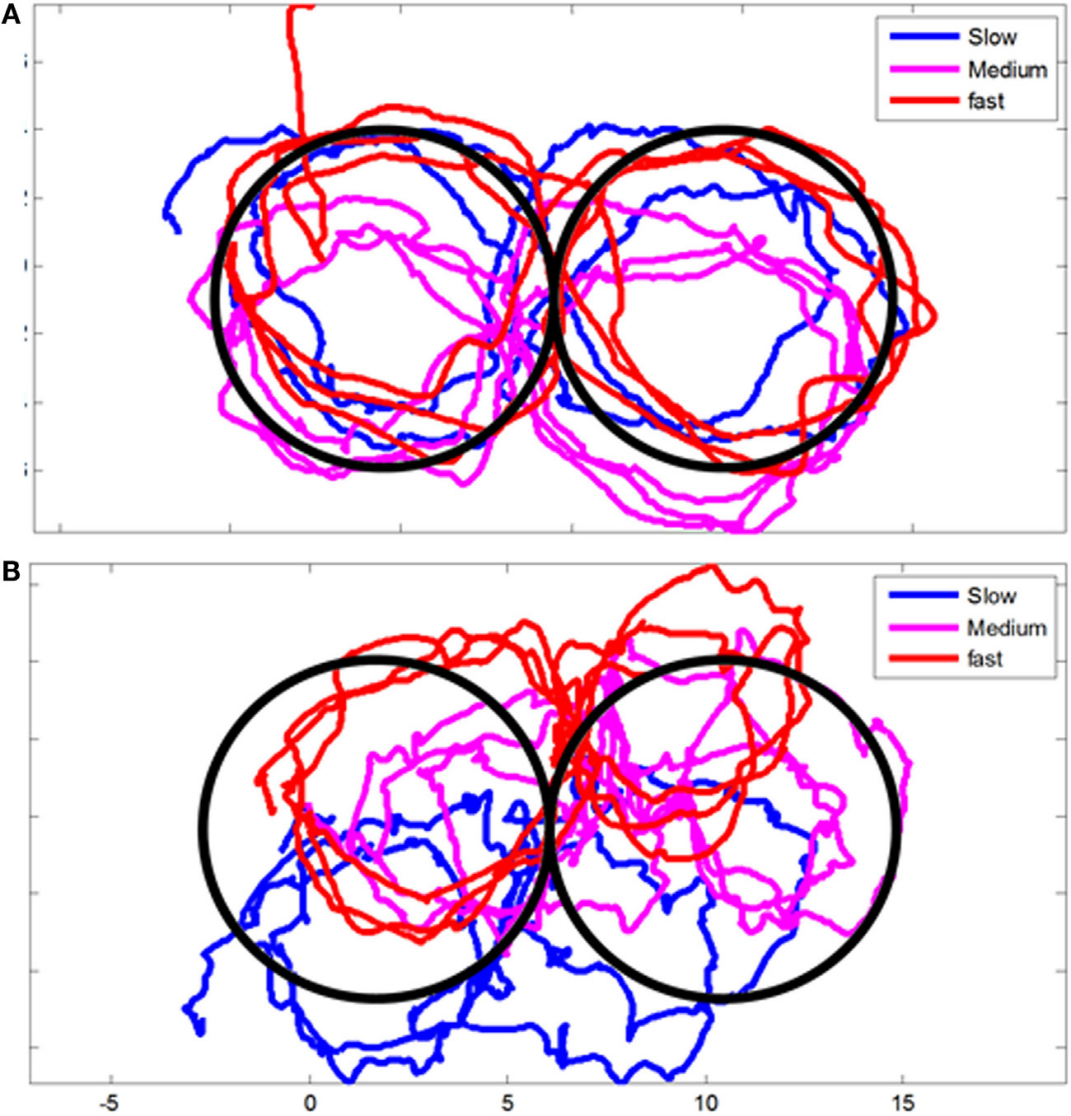
**Example of head movements during figure-eight test**. **(A)** Control volunteer and **(B)** a selected Whiplash patient at early stage displaying impairment of head movement during the test.

Head motion was also recorded while the subjects were asked to trace with their head a figure of eight displayed on the wall at three different paces (slow, moderate, and fast). We could not find a significantly difference between all the patients at early and chronic stages, when we pull together all the results. However, some patient clearly display abnormal head movements during this test as presented in Figure 1. Likewise, we could not find a significantly higher angular RMS velocity between controls and the WAD group at acute and chronic stages.

##### Contribution of Trunk Movements

The amount of trunk movements that subjects performed while tracking a visual target with the laser pointer attached to the head was quantified for two different target movements: linear sinusoidal oscillation in the horizontal and vertical directions and lying-eight-figures, the latter of which were performed at three different speeds.

For the linear tracking motions, we performed a two-factor ANOVA on the normalized contribution of the trunk to tracking movements of the head. We found no main effect of subject group, but we found an interesting and significant interaction between the factors subject group and movement direction. Patients tested shortly after injury moved their trunk less than control subjects for horizontal movements and more than control subjects for vertical movements.

For the figure-8 movements, we performed a three-factor ANOVA with movement speed (slow, medium, and fast) and movement axis (*Y* = horizontal, *Z* = vertical) as within-subject repeated measures and subject group (control, patient – early, patient – +6 months) as a between-subjects factor. The main effect of subject group did not reach significance (*p* = 0.0845), i.e., there was no clear distinction between subject groups for this measure of head vs. trunk movement.

Because of the complex interactions between the speed, axis of rotation and subject-group factors, we computed separate ANOVA for each speed of movement separately, with the *a priori* hypothesis that the fast movements would be most likely to show an effect of subject group. Indeed, there was a significant main effect of subject group computed for the fast speed alone, with no significant cross effect. In other words, the whiplash patients during the horizontal figure eights moved the trunk less than control, similar to the effect we saw during the linear sinusoidal movements. Newman–Keuls *post hoc* tests demonstrated a significant difference between control subjects and patients tested shortly after injury (*p* = 0.001) and a significant difference between the patient groups tested shortly after injury or 6 months after injury (*p* = 0.03), but no significant difference between the control group and the group of patients tested more than 6 months after injury (*p* = 0.11). That is, patients recovered a normal strategy of head trunk coordination.

### Otoneurorogical and Postural Tests

#### Videonystagmography

Abnormal VNG tests (spontaneous or induced nystagmus) were found in four whiplash patients at the early stage. In these four patients, caloric tests were normal. We found any abnormal VNG test at the chronic stage and for the healthy subjects. In summary, the function of horizontal canal function was normal in the majority of the whiplash patients.

#### Cervical VEMP Induced by 500 Hz 102 dB: Study of the Inferior and Mostly Saccular Vestibular Nerve Function

**Figure 2:**
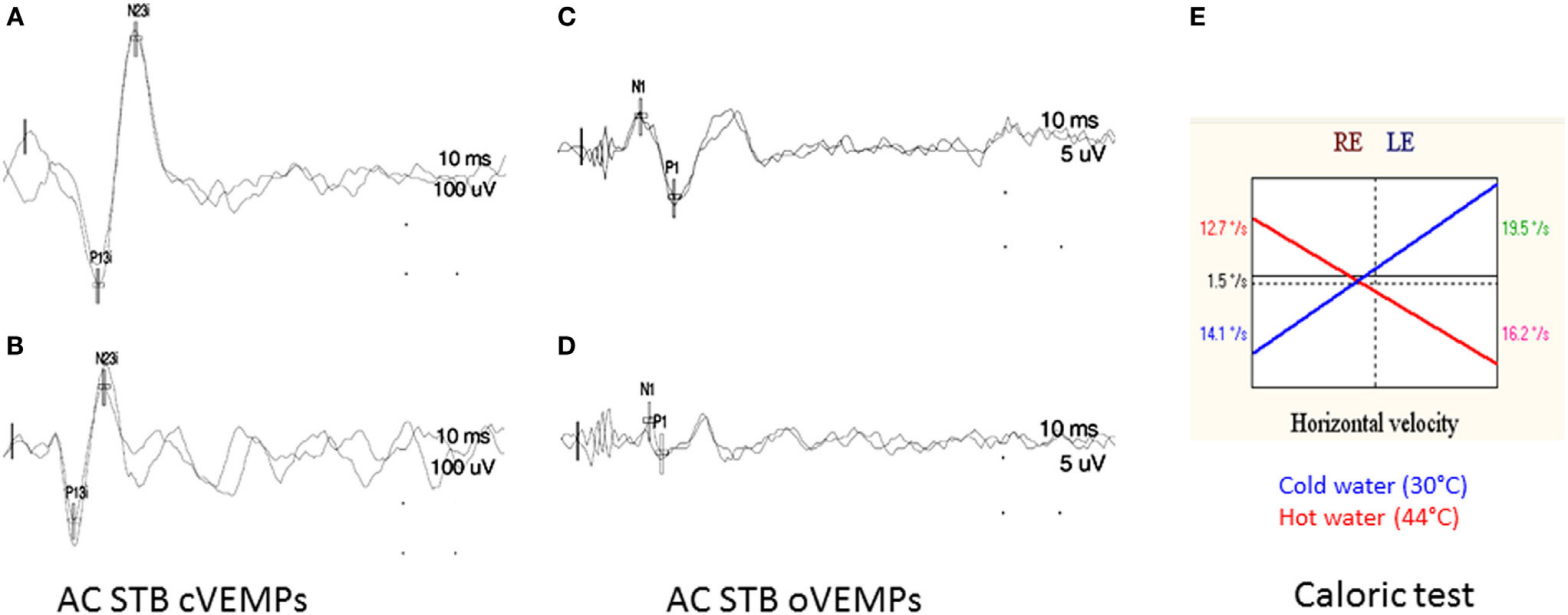
**(A,B) CVEMPs induced by air conducted sound delivered for the right (A) and left (B) ears**. **(C,D)** OVEMPs induced by air conducted sound delivered for the right **(C)** and left **(D)** ears. Note: decreased VEMP’s responses from the left ear compare to the right. **(E)** Illustration of a normal caloric test result.

None of the control group exhibited abnormal cVEMPs to STB and clicks (Figure 2).

#### VEMPs at Early Stage

Twenty-four percent (*n* = 9/37) of the patients/subjects exhibited abnormal 500 Hz STB cVEMPs EPr (decreased *n* = 6 or abolished *n* = 3 on one side; EPr 100). Abnormal cVEMP to high level intensity clicks (105 dB) were found in 28% (*n* = 9/32) of the whiplash patients (Table 2). Five out of 37 patients were NR to high level clicks. They were excluded from the Table 3 because no interesting information could be obtained about the inferior vestibular nerve in these patients.

**Table 3:** **Percentage of normal and abnormal cVEMPs in whiplash patients at early (WAD D15–21) and chronic (WAD M6) stages compared to the Control group**.

	WAD D15–21 (*n* = 37)	WAD M6 (*n* = 17)	Control (*n* = 8)
cVEMP 500 Hz STB abnormal EPr in %	24.3	23.5	0
cVEMP clicks abnormal EPr in %	28.1	14.3	0

#### VEMPs at the Chronic Stage

Approximately 23.5% (*n* = 4/17) showed abnormal cVEMP to STB (decreased *n* = 2 or abolished *n* = 2 on one side) at the chronic stage. 14.3% (*n* = 2/14) exhibited abnormal cVEMPs EPr to high level clicks (Table 3). Three patients were NR to clicks.

#### Comparison of cVEMP at the Early and the Chronic Stages

Cervical vestibular evoked myogenic potentials were tested in 17 patients at both early and chronic stage: 12 were normal at both stage and 5 were abnormal at early stage when the amplitude of P13–N23 was considered. One patient normalized cVEMP to STB and four patients had still abnormal 500 Hz cVEMPs EPr at the chronic stage.

No statistical differences could be detected in the latencies of P13–N23 (Table 4) compared to the ones of our control group (*n* = 8) and our normal (cVEMP) of our cohort group (*n* = 37).

**Table 4:** **Latencies of the P13 and N23 potentials induced by 500 Hz and high level clicks in whiplash patients at early (WAD D15–21) and chronic (WAD M6) stages compared to the control group**.

	Latency P13			Latency N23		
	WAD D15–21	WAD M5	Control	WAD D15–21	WAD M5	Control
cVEMP STB (ms)	14.7 ± 1.3	14.6 ± 1.0	14.6 ± 0.9	21.7 ± 1.6	21.5 ± 1.5	22.3 ± 1.7
cVEMP click (ms)	11.8 ± 1.2	11.6 ± 1.5	11.9 ± 1.07	18.3 ± 1.6	18.1 ± 2.1	18.4 ± 1.8

*Mean ± SD*.

In summary, saccular function was abnormal in average in 23% of the 37 patients at the acute stage. It remained abnormal in 24% of the 17 tested patients at the chronic stage. The decrease of EPr was not associated with abnormal latencies of P13 and N23 potentials.

#### Ocular VEMP Induced by 500 Hz 102 dB: Study of the Superior Vestibular and Mostly Utricular Nerve Function

##### ACS Stimulation

In the healthy group, one subject was NR.

###### Early Stage

*Abnormal ACS oVEMPs to STB were found in 40% (n = 12/30) of the patients, and none in the patients of the control group. Seven out of 30 subjects were bilateraly NRs to ACS*.

###### Chronic Stage

*Approximately 38.4% (n = 5/13) patients had abnormal oVEMPs to ACS. Four patients were classified as NR*.

###### Comparison of oVEMP at the Early and the Chronic Stages

*Seventeen patients were tested at early and chronic stage: 6 were normal at both stages and 5 were abnormal at early stage (2 patients normalized oVEMP to STB and 3 patients had still abnormal 500 Hz oVEMPs at the chronic stage). Six patients were NR at early stage (4 had still no response to STB oVEMPs and 2 had abnormal oVEMPs at the chronic stage)*.

##### BCV Stimulation at Fz

Abnormal Fz oVEMPs were found in 28.5% of the patients at the early stage (*n* = 10/35), in 11.7% (*n* = 2/17) patients at the chronic stage and none in the control group patients. Two patients were NR at the early stage and none at the chronic stage (Table 5).

**Table 5:** **Percentage of normal and abnormal c-oVEMPs clicks in whiplash patients at early (WAD D15–21) and chronic (WAD M6) stages compared to the control group. NR patients to one of the ASC or BCV stimulation were excluded from this table**.

	WAD D15–21 (*n* = 37)	WAD M6 (*n* = 17)	Control (*n* = 8)
oVEMP STB abnormal EPr %	40	38.4	0
oVEMP FZ abnormal EPr %	28.5	11.7	0
oVEMP mastoid abnormal EPr %	22.2	5.8	0

##### BCV Stimulation at the Mastoid Process

Abnormal mastoid oVEMPs were found in 22.2% of the patients at the early stage (*n* = 8/36), 5.8% (*n* = 1/17) patients at the chronic stage and none of the control group. One patient was NR at the early stage and none at the chronic stage (Table 6).

**Table 6:** **Latencies of the n1–p1 potentials clicks in whiplash patients at early (WAD D15–21) and chronic (WAD M6) stages compared to the control group**.

	Latency n1			Latency p1		
	WAD D15–21	WAD M5	Control	WAD D15–21	WAD M5	Control
oVEMP STB (ms)	11.2 ± 0.7	11.2 ± 0.6	10.5 ± 0.4	15.5 ± 1.3	15.3 ± 0.9	14.6 ± 1.0
oVEMP FZ (ms)	11.0 ± 0.8	10.8 ± 0.7	11.2 ± 0.9	15.1 ± 1.0	14.9 ± 0.7	14.7 ± 0.9
oVEMP mastoid (ms)	10.8 ± 0.8	10.7 ± 0.6	10.9 ± 0.8	15.0 ± 1.7	14.9 ± 0.9	15.1 ± 0.8

*Mean ± SD*.

#### Dynamic Posturography Equitest Data

Abnormal scores were found in seven subjects (19%) at the early stage, and none at the chronic stage. Among these, seven patients:

– one whiplash had a lower somesthetic, visual, and vestibular scores;
– three whiplash had lower visual and vestibular scores;
– two whiplash have a lower visual score; and
– one whiplash had a visual preference.

Among the 17 subjects tested at the early and at the chronic stage, 3 (15%) had an abnormal composite score at early stage and their scores returned to normal at the chronic stage.

In summary, equilibrium was abnormal at the early stage in 20% of the patients tested at the early stage. At the chronic stage, all patients had a normal equilibrium function when tested with Equitest.

### Imagery

**Figure 3:**
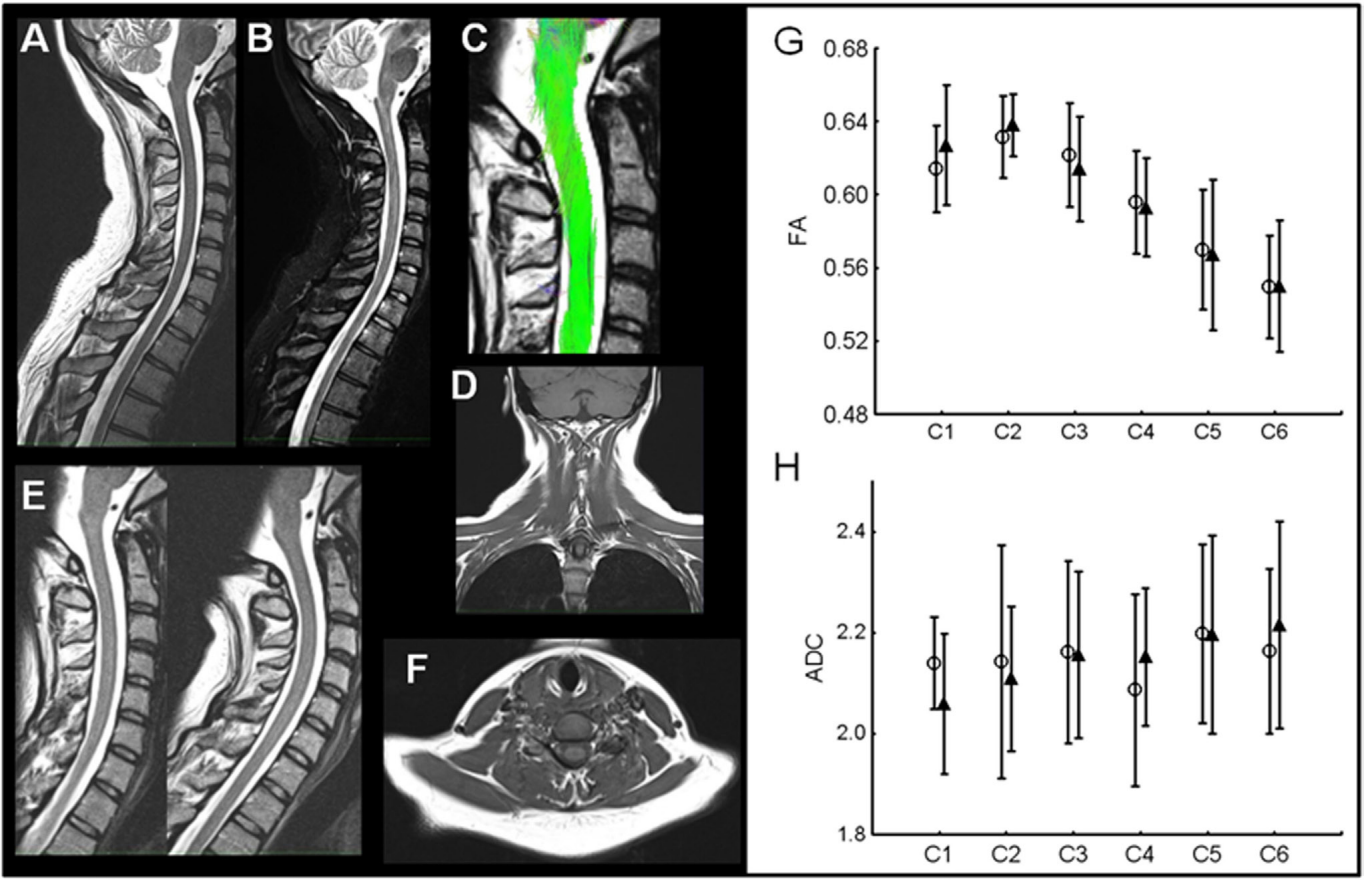
**Example of comprehensive MRI in one WAD patient**. **(A,B)** Sagittal T2and STIR images without any signs of traumatic lesions. **(C)** Spinal cord structural integrity qualitatively similar to control subjects. **(D)** Coronal T1 without traumatic signs in neck musculature. **(E)** Dynamic T2-weighted imaging without any signs of instability. **(F)** Axial T1 images also without traumatic lesions in neck muscles. **(G,H)** DTI group results. Fractional anisotropy (FA) values in WAD patients C1–C6 in acute (open circles) and chronic phase (filled triangles). No difference was present at any cervical level. Apparent diffusion coefficient (ADC) values show no changes with time in WAD patients.

Anatomical MRI did not reveal presence of traumatic lesions in neck musculature (absence of muscle tears and hematoma), bone (no fractures), ligaments, or spinal cord in the patients (Figure 3). Comparison of dynamic MRI did not show any signs of instability: absence of vertebral displacement and disk herniation. DTI-tractography allowed identification of spinal cord fibers from C1 to C6 in all subjects (Figure 3). Quantitative measures of spinal cord structure with DTI showed no change in structure from acute to chronic phase in WAD patients (Figures 3G,H). Measures of AD and RD were also similar between two time points confirming lack of significant structural change in spinal cord at group level.

### Looking for an Early Detection of Poor Outcome after Whiplash

We remind here that patients complaining of pain and psychological sequellae (VAS) at 6 months following the whiplash were labeled as chronic patients (nine patients). Those with no signs at 6 months are called the non-chronic patients (eight patients). The analysis compared the results of a number of tests in the control, chronic and non-chronic group at the early stage. Interestingly, using the QOLIBRI leads to the same partition of patients.

#### Kinematic Head Movement

**Figure 4:**
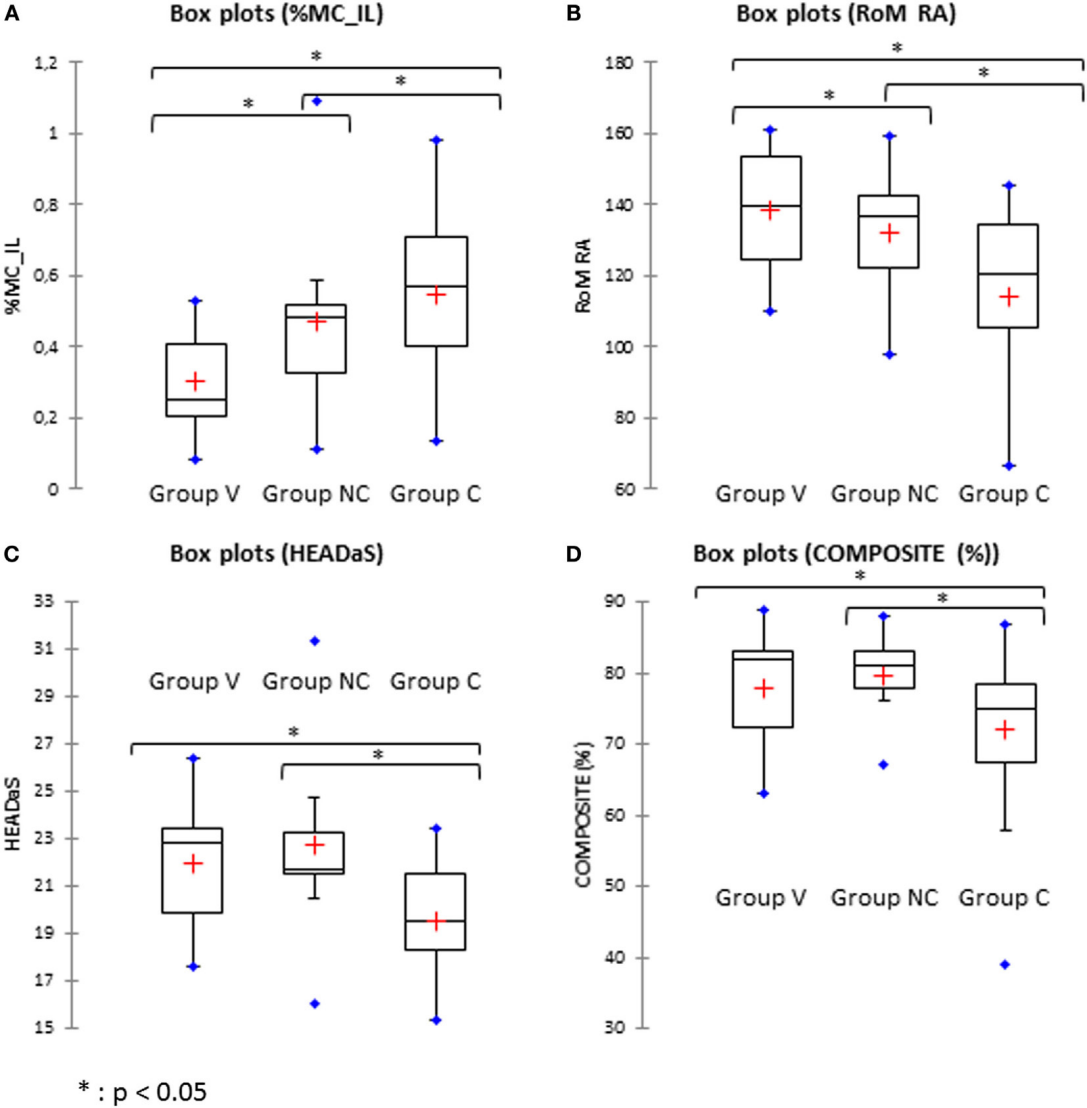
**Comparison between the volunteers (Group V), non-chronic (Group NC), and chronic (Group C) groups**. **(A)** %MC_IL: percentage of coupled movements in lateral bending. **(B)** Rom RA: range of motion in axial rotation. **(C)** HEADaS: mean values of the RMS of the rotational acceleration for the figure-eight pursuit. **(D)** Composite: percentage of composite score for the Equitest^®^.

The range of motion (RoM) in AR and the CM of lateral bending were significantly different between the control, the non-chronic and chronic groups at the early stage (Figures 4A,B). The differences were significantly larger for the chronic group. That is, chronic patients had large reduction of the spontaneous AR and a greater CM during lateral bending of the head.

#### Pursuit

RMS_Yaw, TRUNKaF, and HEADaS were significantly different in chronic patients compare to control. That is, the movement of the trunk during horizontal pursuit (RMS_Yaw) was lower (*p* < 0.05), the trajectory of the trunk (fast trials, *p* < 0.05) and the trajectory of the head (slow trials, *p* < 0.05) during the pursuit were smoother (Figure 4).

#### Vestibular and Postural Tests

The only tests to discriminate chronic patients from control and non-chronic patients were the vestibular composite score of the Equitest (Figure 4). For the oVEMP, both the chronic and the non-chronic patients exhibited significant difference from the control group. When a composite index was built (sum of oVEMP % plus cVEMP %) it was significantly different in chronic and non-chronic patients vs. control.

#### Summary

The kinematics of the head and of the trunk and some parameters of the Equitest were able to discriminate chronic patients from non-chronic one at the early stage following the whiplash. By contrast, vestibular tests were abnormal but unable to discriminate chronic from non-chronic patients.

## Discussion

Early detection of the whiplash patients at risk of developing WAD is important because it would allow designing better preventive treatment for high-risk individual. In order to tackle that issue, we set out to combine several methods of investigations in the same WAD patient at the acute stage of whiplash and we have repeated these observation 6 months later. Several types of investigations were included as follows: a psychologist interviewed participants, a neurologist conducted a detailed clinical examination, and two neurophysiologists submitted the patients to more specialized test concerning gaze and postural control. A neuro-otologist conducted a thorough clinical examination and clinical tests of vestibular function. Finally, the patient underwent CT of the neck, including a DTI study of the descending tract in the spinal cord.

In this study, the pain and/or inflammation of the soft tissues, known as myofascial pain syndrome (MPS), was not took into account. As MPS in whiplash has been already been extensively studied (3, 49–53), this specific aspect should be added in further studies in order to improve the early detection of CWP.

### Neuropsychological Tests

We used the VAS to segregate the patients at 6 months in chronic and non-chronic groups. It could be objected that complain of the patients could not be sufficient to estimate chronicity. In that context, it was important that the QOLIBRI (an estimate of the QoL) and the QDSA (complains), segregated the patients in the same way than the VAS.

Our results showed that high level of catastrophizing and PTSD are predictors of chronic WAD at the early stage. This result is consistent with previous study that investigated PTSD symptoms detrimental influence on the recovery and severity of whiplash complaints following car accidents (54). More specifically, this research showed that the number of hyperarousal symptoms, a dimension of PTSD, at the early stage was found to be related to the persistence and severity of post-whiplash syndrome symptoms at both 6- and 12-month follow-up. Similarly, Kongsted et al. (55) showed that post-traumatic stress response after a whiplash injury was associated with 1-year physical outcome, such as long-lasting pain, poor physical health and reduced working ability. Finally, Sterling et al. (56) reported also that PTSD is a predictor of poor outcome at long-term follow-up. Chronic pain resulting from whiplash injury has often been related to feelings of anxiety. This anxiety is not just a consequence of chronic pain but it can also play an important role in accentuating the sensation of pain (57–59).

Regarding sociologic and demographic variables, our data provide cross results. We failed to find any sex differences while previous studies have observed that being a female is related to poor recovery and persistent disability (60–64). The chronic patients in our research all have a college degree. Thus, these results could not confirm a previous study indicating that WAD patients with a low-educational level report more general distress than patients with a high-educational level (65). Finally, our chronic WAD patients are older than non-chronic patient, which is in accordance with other studies (66–68).

### Motor Control Tests

Eye movements were not recorded in that study, because oculomotor control does not seem deficient in WAD patients (69, 70). By contrast, reduced cervical mobility, disturbed kinesthesia, and altered muscle activity were described at the acute stage of whiplash injuries, and they could persist over time in the moderate/severe groups of patients. In good accordance with these previous studies, our WAD patients exhibited at the acute stage reduced maximal amplitude and abnormal coupling of head movements compared to controls. Even if the choice of the reference frame might have an influence on the calculation of these CMs, this has been considered to have a low influence on the obtained results and on their analysis. Patients complaining of pain had larger loss of AR than pain-free patients. Also, head mobility regained control values, except for ARs in painful patients as described in the Daenen et al. (13).

Whiplash-associated disorder patients were also found to have reduced head smooth pursuit gains compared with healthy controls. They also performed more poorly compared to controls within 1–3 months for the Joint Position Error and Head Repositioning Accuracy Tests. However, these differences were at best small, when significant (31, 71–73). These findings are again in reasonable agreement with our results: we did not find any difference in the amplitude of head pursuit movements between patients and controls for the horizontal movements at the acute and chronic stages, and a small difference for vertical movements, which later disappeared. Also, the NHP test did not show any difference between WAD patients and controls, irrespective of motion direction and of the stage following the lesion. Disappointingly, when the results of the patients where all analyzed together, we could not find either a significantly higher angular RMS velocity between controls and WAD patients at any stages when they performed the figure eight trajectory task proposed by Woodhouse et al. (74). It may be due to our more limited sample of patients, indeed as illustrated in Figure 1, some patients could show abnormal movements. On the other hand, the amount of trunk movements that subjects performed during linear sinusoidal and lying-eight-figures tracking motions differed among controls and WAD patients at the acute stage, which is a new result. Patients tested shortly after injury moved their trunk less than control subjects for horizontal movements and more than control subjects for vertical movements. At the chronic stage, patients recovered a normal strategy of head trunk coordination. An interpretation could be that patients maximize their voluntary head movements during horizontal gaze movement to bypass reflexive gaze control, which could embark them in painful head movements. The same patients would diminish their vertical head movements to minimize pain in the plane where the whiplash occurred. Altogether, neck disturbances combined with dizziness are commonly encountered in the clinic including WAD patients but, the lack of a diagnostic test that establishes that a neck disturbance causes vertigo remains an unanswered question (75).

Studies concerning static postural control in WAD patients have been reviewed by Ruhe et al. (15) and Silva and Cruz (16). They differ in term of type of stance (narrow, comfortable, unipodal, or tandem); visual condition (eyes closed, opened, or visual conflict); type of surface (firm or soft), duration (between 30 and 45 s); heterogeneity in the COP parameters (sway area, displacement, sway energy, stability index, Fourier analysis, and percentage of failure rates in tandem stance). That diversity precluded quantifying the magnitude of the difference of sway between controls and WAD patients. It remains that, out of the 22 studies surveyed in the two reviews, 19 of them revealed that WAD patients exhibited a slight postural instability at rest. Here, we investigated dynamic postural control using the Equitest dynamic posturographic model. Abnormal scores were only found in 20% of the WAD patients at the acute stage, and none at the chronic stage, which is in line with a previous study in 10 WAD patients (76). It suggests that dynamic postural control is also moderately sensitive to whiplash injury.

In summary, we confirm that, at the acute stage, low-grade WAD patients exhibit discrete abnormalities of cervical mobility and postural control. They rarely persist at the chronic stage and their predictive value is poor when considered alone. However, as described below, quantifying abnormalities of cervical mobility and postural control becomes of great values to predict the outcome of the WAD patients, when combined with other symptoms.

### Otoneurorogical Tests

In the present study, the utricular function was abnormal in average in 30% of the 37 patients at the acute stage. It remained abnormal in 30% of the 17 tested patients at the chronic stage. Altogether then, our results are in reasonable agreement with the previous literature on that topic. The review of Tranter and Graham (17) stated that ~10% of patients who have suffered whiplash injury developed otological symptoms. Since then, more specific tests have been used to evaluate the vestibular function in whiplash patients and it may explain the higher percentage of vestibular deficits that we and others had detected in whiplash patients since then. In the study by Solarino et al. (77) based on 14 WAD patients, the cVEMPs displayed decreased amplitude and increased latency and were absent in two patients at 3 months post lesions. While the reduced amplitude could be due a reduction of tonic contraction of SCM muscles to avoid pain, the latency increase was probably related to a lower gain of their vestibulo-collic reflex. In their 2003 study, Ernst et al. (78) investigated 63 patients with vertigo following blunt trauma of the head, neck, and craniocervical junction (without fractures). It included a large proportion of WAD patients. The primary disorders that affected the patients within the first 24 h after trauma included benign paroxysmal positional vertigo (BPPV) (14%), labyrinthine concussion (19%), perilymphatic fistulae (4%), and central vestibular disorders (4%). The following secondary disorders were diagnosed at a later date (3 weeks to 3 months): delayed endolymphatic hydrops (19%), cervicogenic vertigo (27%), and otolith disorders (25%) assessed with cVEMP testing or eccentric rotation. Nacci et al. (79) found that in 90 patients affected with balance disorders following whiplash, VNG tests revealed vestibulopathy in 19% of cases. Finally, Geiger and Aliyev (80) showed that patients submitted to a linear acceleration during the whiplash showed a prevalence of peripheral vestibular, sensory, and vestibulospinal disorders, which is the case of our patients. Those submitted to AR acceleration (absent from our sample) showed a prevalence of central vestibular and sensory disturbances. A single study contrasts with our results: in a retrospective review of 109 patients by Rowlands et al. (81), none reported otological or persistent vestibular symptoms at the acute phase following their whiplash injury. However, these patients were not examined at the chronic stage and apparently not submitted to any vestibular test. Finally, due to our mode of recruitment, none of our patients displayed BPPV although it is known that it can occur in WAD patients (78, 79, 82, 83).

### Imagery

Lesions of various neck tissues (dorsal root ganglia, disks, ligaments, muscles, and vertebral artery) could be instrumental in triggering WAD (84). However, most of the time, these lesions are not detected by imaging techniques and diagnostic tests are not available to assess their clinical relevance. In particular, several publications have used CT and MRI in WAD patients to study the ligamentous structures between the head and the upper cervical vertebrae, with special emphasis on the alar ligament (85–88). The results were not conclusive and the conclusions of the reviews on that topic (20, 89, 90) are straight-forward: at the acute stage of whiplash injury, plain radiography or CT should only be used to exclude fractures and dislocations. MRI of the neck should only be performed when neurological deficits are present. A recent MRI-based studies demonstrated fatty infiltrates in neck muscles of WAD patients (91). They were absent in patients with chronic neck pain and present in WAD patients with higher pain, high disability, and symptoms of PTSD. It suggested that fatty infiltrates may be a meaningful index to detect patients at risk of WAD. Also DTI-tractography had never been used in WAD patients to detect minute lesions of the nervous tract, which could also participate to trigger WAD. In order to test these two hypotheses, we performed a detailed MRI of cervical spinal cord and soft tissues of the WAD patients of our cohort. However, anatomical MRI did not reveal any significant structural change in spinal cord at group level. Therefore, the role of tissue damages in WAD remains to be elucidated and our study does not confirm the usefulness of MRI in chronic WAD patients without neurological deficits.

## Conclusion

As stated in the Section “Introduction,” the mechanisms behind the transition from acute to chronic WAD remain to be elucidated and in particular the evaluation of the impact of multiple risk factors in a single patient. In that context, our study was specifically aimed to combine several methods of investigations in the same WAD patients at the acute stage and 6 months later to investigate that question. Even if our WAD population might be considered small, this is the first study that looked at multi criteria assessment of the risk of chronicity in WAD patients. All chronic patients exhibited high level of catastrophizing at the acute stage and/or PTSD. Their head and trunk motor control values, and in some cases vestibular tests ones, are far from the healthy group. Our “looking for an early detection of poor outcome after whiplash” analysis tested which combination of these symptoms could predict the passage to chronicity in the patients we evaluated test twice over a 6-month period. The outcome is clear and this is the main result of our study: patients displaying high level of catastrophizing at the acute stage and/or PTSD associated with either anomalies in head or trunk kinematics, in the otolithic function and at the Equitest or a combination of these syndromes, turned to chronicity. That is, the association of a neuropsychological disorder with a somatic one was sufficient to explain the passage to chronicity.

Practically speaking, the results of this study are in line with previous results and suggest that low-grade whiplash patients should be submitted as early as possible after the trauma to neuropsychological and motor control tests in a dedicated consultation. In addition, they should be sent to a neuro-otologist for a detailed examination of vestibular functions, which should include cVEMP. Then, if diagnosed at risk of WAD, these patients should be submitted to an intensive preventive rehabilitation program, including vestibular rehabilitation if required. Indeed, it decreases self-perceived handicap and improves postural control in patients with vestibular syndromes (92). Finally, a note of caution should be added. This study concerned a limited number of WAD patients and the analysis was done on uncorrected data. So, to validate the obtained results and to calibrate this proposed approach, a larger multicentric study would be needed in order to build a larger database, including the analysis of other aspects as the myofascial pain.

## Author Contributions

For all the authors: final approval of the version to be published and agreement to be accountable for all aspects of the work in ensuring that questions related to the accuracy or integrity of any part of the work are appropriately investigated and resolved. BS: big work on the review of the paper. SL and P-PV: conception and design of the whole study, general discussion and analysis, and writing of the general draft. DW, JL, and BS: design of the biomechanics and neurophysiological part, acquisition, analysis, and discussion of these results, and writing of this specific part. SB and JA: design of the Neuropsychological part, acquisition, analysis, and discussion of these results, and writing of this specific part. AF and PL: design of the medical imaging part, acquisition, analysis, and discussion of these results, and writing of this specific part. EC and CW: design of the otoneurological part, analysis and discussion of these results, and writing of this specific part.

## Conflict of Interest Statement

The authors declare that the research was conducted in the absence of any commercial or financial relationships that could be construed as a potential conflict of interest.

## Footnotes

^1^https://consult.justice.gov.uk/digital-communications/reducing-number-cost-whiplash/supporting_documents/reducingnumbercostswhiplashclaims.pdf

^2^http://med.inria.fr/

## Abbreviations

AC STB, air-conducted short tone burst; ACS VEMPs, air-conducted sound vestibular evoked myogenic potential; AD, axial diffusivity; ADC, apparent diffusion coefficient; AE, absolute errors; ANOVA, analysis of variance; AR, left–right axial rotation; AWP, acute whiplash patients; BCV, bone-conducted vibration; CNS, central nervous system; CP, canal paresis; CSF, cerebral spinal fluid; CT, computed tomography; CWP, chronic whiplash patients; DTI, diffusion tensor imaging (tractography); ENT, ear nose throat; EPr, evoked potential ratio; FA, fractional anisotropy; FE, flexion–extension; Group C, chronical group; Group NC, non-chronical group; IES-R, impact of events scale-revised; MPS, myofascial pain syndrome; MRI, magnetic resonance imaging; NHP, neutral-head-position; PCS, post-concussion symptom; PTA, pure-tone threshold average; PTSD, post-traumatic stress disorder; QDSA, the Saint-Antoine Pain Questionnaire (Questionnaire Douleur Saint-Antoine); QoL, quality of life questionnaire; QOLIBRI, Quality Of LIfe after Brain Injury; RD, radial diffusivity; RMS, root mean square; RMSE, root mean square error; ROI, region of interest; RoM, range of motion; RTAs, road traffic accidents; SB, left–right side bending; SCM, sternocleidomastoids; STAI, state-trait anxiety inventory; STB, short tone burst; VAS, visual analog scale; VEMP, vestibular evoked myogenic potential; VNG, videonystagmography; WAD, whiplash-associated disorders; Xaj, with j = S for slow, M for medium, F for fast.
